# Prolonged Activation of the Htr2b Serotonin Receptor Impairs Glucose Stimulated Insulin Secretion and Mitochondrial Function in MIN6 Cells

**DOI:** 10.1371/journal.pone.0170213

**Published:** 2017-01-27

**Authors:** Luis Rodrigo Cataldo, María L. Mizgier, Roberto Bravo Sagua, Fabián Jaña, César Cárdenas, Paola Llanos, Dolores Busso, Pablo Olmos, José E. Galgani, José L. Santos, Víctor A. Cortés

**Affiliations:** 1 Department of Nutrition, Diabetes and Metabolism, School of Medicine, Pontificia Universidad Católica de Chile, Santiago, Chile; 2 Institute of Nutrition and Food Technology (INTA), University of Chile, Santiago, Chile; 3 Anatomy and Developmental Biology Program, Institute of Biomedical Sciences, University of Chile, Santiago, Chile; 4 Geroscience Center for Brain Health and Metabolism, Santiago, Chile; 5 Buck Institute for Research on Aging, Novato, CA, United States of America; 6 Institute for Research in Dental Sciences, School of Odontology, University of Chile, Santiago, Chile; 7 UDA-Health Sciences, Nutrition and Dietetic Program, School of Medicine, Pontificia Universidad Católica de Chile, Santiago, Chile; Broad Institute, UNITED STATES

## Abstract

**Aims:**

Pancreatic β-cells synthesize and release serotonin (5 hydroxytryptamine, 5HT); however, the role of 5HT receptors on glucose stimulated insulin secretion (GSIS) and the mechanisms mediating this function is not fully understood. The aims of this study were to determine the expression profile of 5HT receptors in murine MIN6 β-cells and to examine the effects of pharmacological activation of 5HT receptor Htr2b on GSIS and mitochondrial function.

**Materials and Methods:**

mRNA levels of 5HT receptors in MIN6 cells were quantified by RT qPCR. GSIS was assessed in MIN6 cells in response to global serotonergic activation with 5HT and pharmacological Htr2b activation or inhibition with BW723C86 or SB204741, respectively. In response to Htr2b activation also was evaluated the mRNA and protein levels of PGC1α and PPARy by RT-qPCR and western blotting and mitochondrial function by oxygen consumption rate (OCR) and ATP cellular content.

**Results:**

We found that mRNA levels of most 5HT receptors were either very low or undetectable in MIN6 cells. By contrast, Htr2b mRNA was present at moderate levels in these cells. Preincubation (6 h) of MIN6 cells with 5HT or BW723C86 reduced GSIS and the effect of 5HT was prevented by SB204741. Preincubation with BW723C86 increased PGC1α and PPARy mRNA and protein levels and decreased mitochondrial respiration and ATP content in MIN6 cells.

**Conclusions:**

Our results indicate that prolonged Htr2b activation in murine β-cells decreases glucose-stimulated insulin secretion and mitochondrial activity by mechanisms likely dependent on enhanced PGC1α/PPARy expression.

## Introduction

Although most studies on serotonin (5-hydroxytryptamine, 5HT) focus on its role as a central neurotransmitter, the bulk of 5HT (>90%) is synthetized by enteroendocrine cells, secreted to systemic circulation and stored in platelets [1]. Additional peripheral tissues are also able to synthesize and release small amounts of 5HT [1, 2]. These microserotoninergic systems have been involved in auto and paracrine regulatory circuits. Nowadays, pancreatic β-cells are recognized as a *bona fide* microserotonergic system, able to synthesize, store and release 5HT in response to glucose stimulation [3–6]. Whether 5HT has physiological or pathophysiological implications on glucose-stimulated insulin secretion (GSIS) is still controversial.

Current evidence indicates that extracellular 5HT modulates GSIS depending on the specific 5HT receptors expressed by β-cells as well as the extent of serotoninergic stimulation [7–9]. Nonetheless, a systematic exploration of the identity and function of 5HT receptors in pancreatic β-cells is still lacking [10]. Recent studies have shed light on the role of 5HT receptors in GSIS. Gene deletion of ionotropic Htr3a receptor results in gestational diabetes in mice [8] and glucose intolerance upon feeding a high-fat diet [9]. Accordingly, acute Htr3 stimulation increases GSIS in states of high metabolic demand such as pregnancy and obesity [8, 9]. By contrast, prolonged exposure of pancreatic β-cells and murine islets to 5HT decreases GSIS [11, 12]. This effect is replicated by prolonged pharmacological activation of Htr2c receptor in mouse pancreatic islets and MIN6 cells [13]. Nonetheless, although Htr2b receptor mediates insulin opposite actions of gut-derived 5HT in adipocytes and hepatocytes [14, 15], the role of Htr2b in β-cells regulating GSIS has been poorly studied.

Pancreatic β-cells requires normal mitochondrial function for glucose stimulated ATP production and insulin secretion [16, 17]. PPARy co-activator 1α (PGC1α) is a key regulator of mitochondrial biogenesis. Although its exact role in β-cells function has not been elucidated, PGC1α seems to negatively affect the β-cell differentiation and insulin secretion. Fetal PGC1α overexpression determines pancreatic β-cell dysfunction during the adulthood through inhibition of Pdx1 expression [18] and the overexpression of PGC1α in pancreatic islets decreases GSIS in rats [19]. Interestingly, PGC1α levels are higher in pancreatic islets of diabetic mice in comparison with non-diabetic animals [19], suggesting a role for excessive PGC1α activity on beta cell dysfunction in diabetes.

It has been shown that the pharmacological activation of Htr2 receptors increases the transcriptional activity of PGC1α promotor and its mRNA and protein expression levels in epithelial renal cells [20]. Furthermore, overexpression of Htr2b in heart of mice leads to abnormal mitochondrial function in cardiomyocytes [21]. Whether prolonged Htr2b activation in β-cells impairs GSIS and what are its effects on mitochondrial functioning and PGC1α levels remains unknown.

Herein, we show that 5HT receptor Htr2b is expressed in MIN6 β-cells and mouse pancreatic islets and its prolonged activation decreases GSIS in association with increased PGC1α expression and decreased mitochondrial function.

## Material and Methods

### MIN6 cells

MIN6 cells, a mouse pancreatic β-cell line stablished by Miyazaki et al. [22] were donated by Dr. Francisco Pérez-Bravo (University of Chile, Santiago, Chile). Cells were maintained at 37°C in a 5% CO_2_ atmosphere in Dulbecco’s modified Eagle medium (DMEM) containing 10% fetal bovine serum (FBS), 25 mmol/l glucose, 3.7 g/L sodium bicarbonate, 100 U/ml penicillin and 100 mg/ml streptomycin. All culture solutions were purchased from Thermo Fisher Scientific (Waltham, MA).

### GSIS assay

MIN6 cells and pancreatic islets were incubated in glucose-free or low glucose (2.8 mM) Krebs-Ringer HEPES buffer (KRH; NaCl 130 mmol/L, KH_2_PO_4_ 1.25 mmol/L, MgSO_4_ 1.25 mmol/L, CaCl2 2.68 mmol/L, NaHCO_3_ 5.26 mmol/L, HEPES 10 mmol/L) respectively, for 30 min, to increase glucose sensitivity. Then, cells and islets were incubated with glucose-free or low glucose (2.8 mM) and with high-glucose (20 or 16.7 mM) KRH buffer 0.5% BSA, respectively, for 1 h. Assay buffers were then centrifuged for 10 min at 5000 rpm at 4°C and stored at –20°C. Insulin was measured with mouse/rat insulin ELISA kit (Merck Millipore, Billerica, MA). GSIS in cells and islets was expressed as insulin concentration normalized to total cellular proteins or total insulin content, respectively, and relativized as the Stimulation Index (SI). GSIS in MIN6 cells was repeated three times independently and each measurement was performed in triplicate. GSIS in mice islets was performed only once in a single experiment with 5 islets per well/condition. Although the measurement of each was replicated four times we present the data as a single value, corresponding to the average of these determinations.

### 5HT measurement

MIN6 cells were incubated with 5HT precursor 5-hydroxytryptophan (5HTP) (500 μM) or vehicle in DMEM medium without FBS for 6 h and then 5HT concentration was quantified in the conditioned medium by HPLC. Proteins in the media were precipitated with 3.4 M perchloric acid and centrifuged at 13,000 rpm for 5 min. 20 μl of supernatant were used for HPLC analysis. The HPLC system consisted in an Ultrasphere 5-μm ODS column (HiChrom, Theale, UK), Rheodyne manual injector (Sigma-Aldrich, St. Louis, MO), and Waters 515 HPLC pump (Waters, Milford, MA). HPLC mobile phase was 0.1 M sodium acetate at 1 ml/min. Detection was performed with a Waters 464 electrochemical detector set at 500 mV, current 10 nA and latency of 5 s, using EMPOWER software (Waters, Milford, MA) for analysis. 5HT mass was estimated by interpolation into a calibration curve.

### Animals

Wild-type and *db/db* mice (C57BL6/J genetic background in both cases) were obtained from Jackson Laboratories (Bar Harbor, ME) and housed in colony cages (3–4 per cage). Animals were maintained on a 12 h light cycle with controlled humidity and temperature and with free access to standard chow diet and water. Animal procedures were approved by the Bioethical and Animal Welfare Committee from the School of Medicine, Pontificia Universidad Católica de Chile (Permit Number 14–011).

### Pancreatic islets isolation

Only male 16–20 weeks old WT (24–30 g) and *db/db* (55–60 g) mice were used in this study. Pancreatic islets were isolated as previously reported [23, 24]. Briefly, animals were sacrificed with ketamine/xylazine overdose (100/10 mg/kg) and the pancreas was digested *in situ* by perfusion with 0.21 mg/ml Liberase TL Research Grade collagenase (Roche, Basel, Switzerland). Pancreas from three mice were pooled in every islets isolation assay and further incubated for 14 min at 37°C with collagenase solution. The digestion was stopped with RPMI medium 10% FBS. After rinsing steps, the tissue suspension was filtered through a 250 μm wire mesh and isolated islets were separated by Histopaque1077 (Sigma-Aldrich, St. Louis, MO). Based on morphological criteria, healthy islets were manually selected for both GSIS assays and gene expression profiling. Islets used for RNA extraction were stored in Ambion PureLink lysis buffer (Thermo Fisher Scientific, Waltham, MA) at -80°C.

### mRNA levels analysis

Total RNA was extracted from MIN6 cells or pooled mice islets using Ambion PureLink affinity columns (Thermo Fisher Scientific, Waltham, MA). For 5HT receptors genes, cDNA was obtained from 600 ng of RNA using RT2 first strand system and analyzed with a RT-Profiler Custom PCR-Array (Qiagen, Hilden, Germany). For PGC1α, PPARy and mitochondrial complexes mRNA analysis, cDNA was obtained from 1000 ng of RNA using AffinityScript qPCR cDNA Synthesis Kit (Agilent Technologies, Santa Clara, CA) and amplified with previously validated primers [25] (S1 Table). Gene expression in MIN6 cells was analyzed from three independent experiments. The relative abundance of 5HT receptors mRNA was expressed as 2^−ΔCt 5HT receptors^/2^−ΔCt InsR^, where ΔCt = average Ct of target genes (5HT receptors or insulin receptor) minus the average Ct of housekeeping genes β-Actin, Glyceraldehyde-3-phosphate dehydrogenase and β-Glucuronidase. InsR was selected as a comparator because the absolute abundance of its mRNA was similar (Ct = 25) with some 5HT receptors evaluated in the PCR-array. For gene expression analysis in mice pancreatic islets, the relative mRNA abundance was expressed as 2^−ΔCt *db/db*^/2^−ΔCt WT^.

### PGC1α overexpression

400,000 MIN6 cells/well were seeded in 6-well plates and maintained for 48 h to reach ~70% confluence. Cells were transiently transfected with 3 μg of plasmid DNA (pCDNA3.1-PGC1α or pCDNA3.1-mock) using Lipofectamine 2000, following manufacturer´s recommendations (Thermo Fisher Scientific, Waltham, MA).

### Immunoblot

MIN6 cells were washed with cold PBS (pH 7.4) and lysed with RIPA buffer containing protease inhibitor cocktail (Thermo Fisher Scientific, Waltham, MA). Lysates were centrifuged at 14,000 g for 15 min at 4°C, and the supernatant was stored at –20°C. Protein concentration was quantified with Pierce BCA Protein Assay Kit (Thermo Fisher Scientific, Waltham, MA). 20–30 μg of the protein extracts were denatured with Laemmli buffer (SDS 10%, glycerol 50%, DTT 6 M and bromophenol blue 1.2%), SDS-PAGE separated and electrotransferred onto nitrocellulose membranes (350 mA for 60 min). Membranes were blocked with 5% fat-free milk in TBS-T buffer (50 mM Tris-HCl, pH 7.6, 150 mM NaCl, 0.1% Tween 20) for 90 min and incubated overnight at 4°C with primary antibodies. A primary antibody against Htr2b (1:1000) was obtained from Aviva Systems Biology (code: OAAB07368). Antibodies against PGC1α (1:500), Gapdh (1:600) and histone H3 (1:1500) were obtained from Santa Cruz Biotechnology (codes: sc-13067, sc-25778 and 4499P, respectively). After rinsing with TBS-T, membranes were incubated with a HRP-conjugated anti-rabbit IgG secondary antibody (code: 7074; Cell Signaling, Danvers, MA) for 60 min. Immunoblots were visualized with Pierce ECL kit (Thermo Fisher Scientific, Waltham, MA). Intensity of bands was quantified with ImageJ software (NIH, https://imagej.nih.gov/ij).

### ATP quantitation

MIN6 cells were lysed with ice-cold reaction buffer (200 mM Tris, pH 7.5; 2 M NaCl; 20 mM EDTA; 0.2% Triton X-100). Total ATP content was quantified with ATP Determination Kit (Thermo Fisher Scientific, Waltham, MA). Protein concentration was determined with Pierce BCA Protein Assay Kit for normalization.

### Mitochondrial respiration assay

Mitochondrial oxygen consumption rate (OCR) was evaluated with XFe96 extracellular flux analyzer (Agilent Technologies, CA, USA). MIN6 cells were seeded at 40000 cells/well on 96-well XFe96 cell culture microplates and cultured for 48 h. Cells were treated with Htr2b selective agonist BW723C86 (10 μM) or vehicle (DMEM) for 6 h. For respiration assay, cells were incubated in a CO_2_-free environment for 1 h and OCR was measured every 3 minutes for the next 90 min. First, OCR was quantified in basal condition (20 mM glucose), then with 1 μM oligomycin (ATP Synthase inhibitor), then with 0.125 μM FCCP (mitochondrial respiration uncoupler), and finally with 1 μM Rotenone/Antimycin A (Complex I and III inhibitors, respectively). OCR data was normalized to total protein content and analyzed with Wave Seahorse Software. Non-mitochondrial respiration was subtracted to the other parameters.

### Intracellular calcium imaging

400,000 MIN6 cells/well were seeded on glass coverslips in 6-well plates, then loaded for 30 min at 37°C with 5 μM Fluo-4-AM (Thermo Fisher Scientific, Waltham, MA) dissolved in glucose-free KRH buffer with 0.02% Pluronic acid. After washing, intracellular calcium was recorded with an Axiovert 200 LSM 5 Pascal confocal microscope (Carl Zeiss, Oberkochen, Germany). Basal fluorescence was recorded for 120 s. Afterward, the cells were stimulated with 10 μM BW723C86. As a positive control, MIN6 cells were treated with 30 μM Carbachol. Different regions of interest (ROI) were analyzed for each experimental condition.

### Mitochondrial DNA copy number

Mitochondrial DNA copy number was estimated as previously described [26]. Briefly, MIN6 cells were seeded at 400,000 cells/well on 6-well plates and maintained for 48 h. Following experimental treatment, cells were washed with cold PBS (pH 7.4) and total DNA was extracted with QIAamp DNA Blood kit (Qiagen, Hilden, Germany). The copy number of mitochondrial gene *ND5* and nuclear gene *GAPDH* was quantified in 50 ng of total DNA by real-time PCR using the following primers: *ND5*, forward primer: 5´-CTGGCAGACGAACAAGAC-3´, reverse primer: 5´-GAGGCTTCCGATTACTAGG-3; *GADPH*, forward primer: 5´-CAATGTGTCCGTCGTGGATCT-3´, reverse primer: 5´-GTCCTCAGTGTAGCCCAAGAT-3´. Relative quantification of ND5/GAPDH was calculated by the ΔCt method (ΔCt = Ct_ND5_ –Ct_GAPDH_). Final result was multiplied by 2 (2·2^-ΔCt^), due to the diploid abundance of *GAPDH*.

### Mitochondrial superoxide quantitation

40,000 MIN6 cells/well were seeded in a black 96-well plate and maintained in standard culture conditions for 48 h. Cells were treated with Htr2b selective agonist BW723C86 (10 μM) or vehicle (DMEM) for 6 h. Cells were washed and incubated for 30 min with glucose-free KRH buffer. Later, cells were incubated for 15 minutes with 20 mM glucose KRH buffer supplemented with 5 μM MitoSOX (Thermo Fisher Scientific, code: M36008). After rising with glucose-free KRH, cellular fluorescence (excitation 530/25 nm and emission 590/35 nm and a set gain of 80) was quantified with a microplate reader (Biotek, Synergy HT. Afterward, cells were lysed and whole cell protein concentration was measured with Pierce BCA Protein Assay. Relative fluorescence units (RFU) were normalized to total protein content (RFU/mg of protein).

### Statistical analysis

Data of studies with MIN6 cells was expressed as mean ± standard error (SEM) of at least 3 independent experiments. Student's t test or one-way ANOVA were used for simple or multiple comparisons, respectively. Statistical significance was set at p < 0.05. Data of studies with mice pooled pancreatic islets is expressed as the mean of 3 or 4 replicated determinations. Prism 6.0 (Graph Pad) was used for statistical analysis and graphics generation.

## Results

### MIN6 cells secrete 5HT to the extracellular medium and exogenous 5HT decreases GSIS

**Fig 1:**
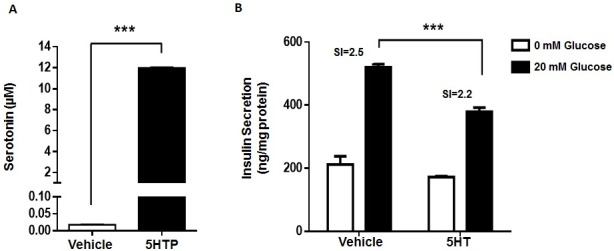
5HTP increases extracellular 5HT levels in MIN6 cells and 5HT reduces GSIS. (A) MIN6 cells were incubated with 5HT precursor 5HTP (500 μM) (black bars) or vehicle (white bars) for 6 hours and then 5HT concentration was quantify in the conditioned medium by HPLC. (B) MIN6 cells were incubated with 5HT (50 μM) or vehicle during 6 hours in DMEM without FBS and then subjected to GSIS procedure with 0 (white bars) or 20 mM glucose (black bars) for 1 hour. Insulin concentration in the KRH buffer was quantified by ELISA. Graph bars represent mean ± SEM of measurements of three independent experiments in triplicate. The symbol *** denotes p<0.001 in Student’s t (A) tests and in one-way ANOVA (B).

We have reported that MIN6 cells express microserotoninergic genes at the mRNA level and that can synthesize and degrade 5HT [5, 6] and that pre-incubation with the immediate serotonin (5HT) precursor biosynthetic 5HTP decreases GSIS in MIN6 cells [5]. Herein, we show that pre-incubation with 5HTP for 6 h increases 5HT levels in the culture medium of MIN6 cells (Fig 1A) and that pre-incubation with 5HT for 6 h decreases GSIS Fig 1B). Therefore, MIN6 cells, a well recognized model for β-cells, are able to synthesize, and release 5HT, and extracellular 5HT decreases GSIS in these cells.

### Htr2b is present in MIN6 cells and in pancreatic islets of wild type and *db*/*db* mice

**Fig 2:**
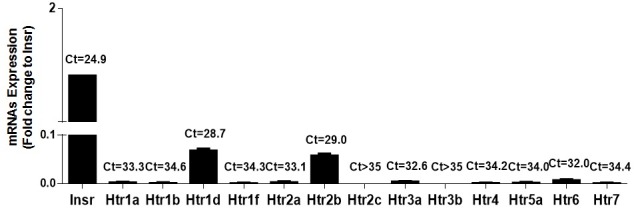
5HT receptors mRNA levels in MIN6 cells. Total RNA was extracted from MIN6 cells and 5HT receptors mRNA levels assessed by RT-qPCR. The mRNA levels was expressed as fold-change to Insulin receptor (Insr) (2^−ΔCt 5HT receptors^/2^−ΔCt Insr^). Bars correspond to mean ± SEM of three independent experiments. Numbers over the bars correspond to mean Ct values.

To explore the mechanisms by which extracellular 5HT decreases GSIS, we quantified the expression level of thirteen 5HT receptors in MIN6 cells. Most 5HT receptors were very low or undetectable at mRNA level (Fig 2). In fact, only Htr1d and Htr2b were detected with CT values <30 (Fig 2). Htr2b was also present at protein level and tend to be up-regulated after treatment with BW723C86, a selective Htr2b agonist, in MIN6 cells (S1 Fig).

To assess the physiological and pathological implications of 5HT receptors on GSIS *in vivo*, we quantified 5HT receptors in pancreatic islets of wild type (WT) and *db/db* mice, a model of obesity-induced diabetes with β-cells dysfunction [27]. The mRNA level of Htr2b was detected at low level in islets of WT mice, but its level was around 3 fold higher in islets of *db/db* mice (S2 Table), suggesting that Htr2b may be implicated in decreased GSIS in this diabetes mouse model.

Given the presence of Htr2b in MIN6 cells, as well as, in islets from WT and *db*/*db* mice, we evaluated the role of this receptor on GSIS.

### Prolonged Htr2b activation decreases GSIS in MIN6 cells

Htr2b is a Gα_q_-coupled receptor that activates phospholipase C (PLC)/IP3 pathway and thereby promotes intracellular calcium elevation [7, 28]. As expected, acute stimulation with BW723C86, increased intracellular calcium levels in MIN6 cells (S2 Fig), suggesting that Htr2b is functionally coupled to PLC/IP3 pathway in these cells.

**Fig 3:**
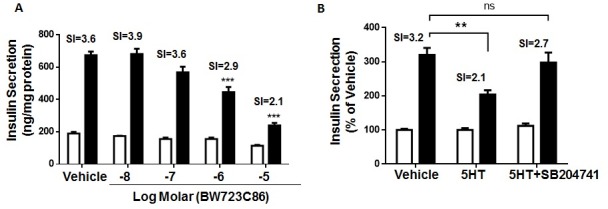
Prolonged activation of Htr2b reduces GSIS in MIN6 cells. (A) MIN6 cells were treated with vehicle or increasing concentrations (10 nM– 10 μM) of the selective Htr2b agonist BW723C86 for 6 hours and then subjected to GSIS procedure. (B) MIN6 cells were treated with vehicle, 50 μM 5HT or co-treated with 5HT (50 μM) plus a selective Htr2b antagonist SB204741 (100 nM) for 6 hours and then subjected to GSIS procedure. The GSIS assays were carry out with KRH buffer 0 (white bars) or 20 mM glucose (black bars). Concentration of insulin accumulated over 1 hour was measured by ELISA. The bars represent mean ± SEM of three independent experiments in triplicate. Number over the bars represents the stimulation index (SI). ** p<0.01, *** p<0.001 and ns, non-significant. One-way ANOVA analysis.

To determine the effects of Htr2b activation on GSIS, MIN6 cells were incubated for 6 h with BW723C86. As shown in Fig 3A, BW723C86 decreased GSIS in MIN6 cells with a half-maximal inhibitory concentration (IC_50_) of 1.3 μM (Fig 3A). A similar response was observed in pancreatic islets from WT mice (S3 Fig). To further evaluate the role of Htr2b on 5HT-dependent GSIS inhibition, MIN6 cells were co-treated for 6 h with 5HT and Htr2b specific antagonist SB204741. As shown in Fig 3B, 100 nM SB204741 prevented 5HT-induced GSIS reduction, suggesting that 5HT-mediated activation of Htr2b is responsible for GSIS impairment in MIN6 cells.

### Prolonged Htr2b activation increases PGC1α and PPARy levels in MIN6 cells

Mitochondrial glucose oxidation is required for normal insulin secretion [29] and PGC1α is a key regulator of mitochondrial biogenesis [30]. Elevated levels of nuclear receptor PPARy have been associated with impaired insulin secretion [31] and treatment with PPARy pharmacological activators reduces GSIS in cultured β-cells [32]. In a renal tubular cell line, non-selective pharmacological activation of Htr2 receptors increases PGC1α levels [20]. Therefore, we evaluated the effects of prolonged activation of Htr2b on PGC1α and PPARy levels in MIN6 cells.

**Fig 4:**
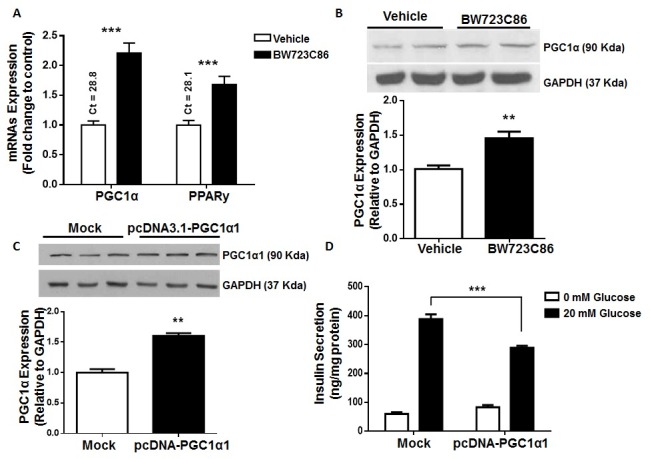
Prolonged Htr2b activation increases PGC1α and PPARy levels in MIN6 cells and the effect of PGC1α1 overexpression on GSIS. (A) MIN6 cells were treated for 6 hours with the Htr2b selective agonist BW723C86 (10 μM) and then the mRNA levels of PGC1α and PPARy were quantified by RT-qPCR. Relative mRNA levels are expressed as fold-change of 2^−ΔCt^ in treated vs. control cells (2^−ΔCt treated^/2^−ΔCt control^). Numbers over bars correspond to mean Ct values. (B) MIN6 cells were treated for 24 hours with BW723C86 (10 μM) and then, PGC1α and Gapdh protein expression was evaluated by immunoblot. (C and D) MIN6 cells were transfected with a pcDNA3.1-PGC1α1 or an empty vector (mock) for 48 hours. (C) Protein levels of PGC1α1 and (D) insulin secretion were measured by immunoblot and GSIS procedure, respectively. Graph bars represent means ± SEM of three independent experiments in triplicate. ** p<0.01 and *** p<0.001 in one-way ANOVA analysis (A and D) or Student’s t test (B and C).

As shown in the Fig 4A, pre-incubation with BW723C86 for 6 h significantly increased PGC1α and PPARy mRNA levels in these cells. At the protein level, BW723C86 significantly increased whole cell content of PGC1α (Fig 4B). An equivalent finding was observed in nuclear PGC1α content (S4 Fig), suggesting that Htr2b activation increases PGC1α levels and possibly its transcriptional activity in MIN6 cells. Importantly, the role of PGC1α on GSIS remains controversial [18, 19, 33, 34]. We evaluated the effects of increased PGC1α levels on GSIS in MIN6 cells.

### Overexpression of the PGC1α decreases GSIS in MIN6 cells

The four known isoforms of PGC1α have different molecular and cellular actions [25], thus we explored the expression profile of PGC1α isoforms in MIN6 cells and mouse pancreatic islets. At the mRNA level, PGC1α1 was the most abundant isoform (CT<30) in MIN6 cells (S5A Fig). Equivalent findings were observed in pancreatic islets of WT and *db*/*db* mice (S5B Fig).

To determine the effects of elevated PGC1α on GSIS, MIN6 cells were transiently transfected with an expression plasmid encoding PGC1α1. As shown in Fig 4C and 4D, PGC1α1 overexpression decreased GSIS in MIN6 cells, suggesting that Htr2b-dependent elevation of PGC1α result in impaired GSIS in these cells.

### Prolonged activation of Htr2b impairs mitochondrial activity in MIN6 cells

**Fig 5:**
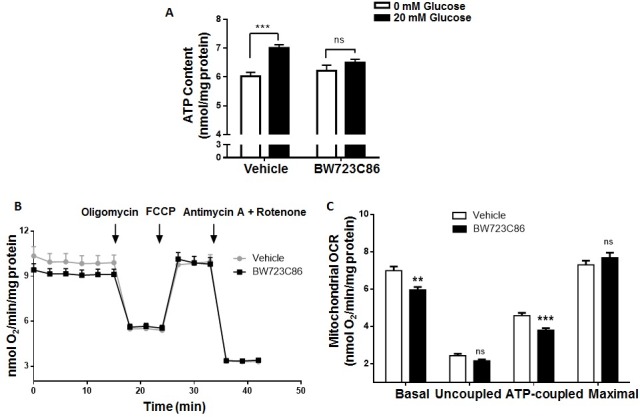
Prolonged Htr2b activation decreases ATP content and coupled mitochondrial respiration in MIN6. (A) MIN6 cells were treated for 6 hours with Htr2b selective agonist BW723C86 (10 μM) or vehicle and then subjected to GSIS. Total ATP content was quantified by a Luciferase assay and normalized to the total protein content of cells stimulated by 0 (white bars) or 20 mM glucose (black bars). (B-C) MIN6 cells were treated by 6 hours with BW723C86 (10 μM) or vehicle and then the oxygen consumption rate (OCR) was measured. (B) OCR in cells after the successive addition of glucose (20 mM), Oligomycin (1 μM), FCCP (0.125 μM) and Antimycin A (1 μM) plus Rotenone (1 μM). (C) The bars represents the mitochondrial basal, uncoupled, ATP-coupled and maximal OCR adjusted by total protein content in BW723C86 treated (black bars) and control (white bars) MIN6 cells. Graph bars represent means ± SEM of three independent experiments. ** p<0.01 and *** p<0.001, ns, non-significant in one-way ANOVA.

PGC1α overexpression blunts glucose-induced ATP production in rat islets [19]. Thus, we evaluated whether prolonged activation of Htr2b for 6 h changes total ATP content and mitochondrial activity after glucose stimulation in MIN6 cells. As shown in Fig 5A, glucose stimulation increases ATP content by 16% in MIN6 cells and this was completely prevented by BW723C86.

We quantified oxygen consumption rate (OCR) in MIN6 cells after incubation with BW723C86 for 6 h. Fig 5B shows the changes in OCR in MIN6 cells upon a protocol for cellular respiration evaluation. After subtracting non-mitochondrial respiration (defined as the OCR following Antimycin A/Rotenone treatment), basal, uncoupled, ATP-coupled and maximal mitochondrial respiration rates were determined. OCR analysis showed that prolonged treatment with BW723C86 significantly reduced basal and ATP-coupled mitochondrial respiration in MIN6 cells (Fig 5C), suggesting that reduced GSIS after Htr2b activation may be the result of impaired mitochondrial function and blunted raise in ATP content in response to glucose stimulation.

Importantly, we did not observe changes in mitochondrial superoxide (31.0±0.5 vs. 30.2±1.4 RFU/mg of protein, p = 0.79), DNA copy number (0.9±0.1 fold change, p = 0.42) and UCP2 mRNA levels (1.2±0.3 fold change, p = 0.40) in MIN6 cells after prolonged pre-incubation with BW723C86. These results are consistent with the unchanged uncoupled and maximal mitochondrial respiration after BW723C86 in MIN6 cells.

We found no changes in the mRNA levels of Ndufa1, Sdha, Cox5a, genes encoding components of complexes I, II and IV, respectively (S6 Fig). By contrast, complex V components Atp4a and Atp5a1 significantly decreased and increased, respectively, at the mRNA level in MIN6 cells after BW723C86 treatment (S6 Fig).

## Discussion

We previously found that MIN6 cells as well as islets from WT and *db/db* mice express several serotonergic genes, including those encoding for 5HT biosynthetic enzymes Tph1/2 and Ddc, vesicle transporters Vmat1/2 and catabolic enzymes Maoa/b [5, 6]. Others have described equivalent findings in mouse islets [3, 4]. Additionally, in this and previous work [5], we have shown that 5HT precursor 5HTP and Mao inhibitor Pargyline, increases 5HT content and extracellular 5HT levels in MIN6 cells, indicating these cells synthesize, degrade, store and secrete 5HT, constituting a functional microserotonergic system.

In our previous work [5] as well as in this current manuscript, we report that 5HTP pre-incubation increases extracellular 5HT in MIN6 cells and that it is associated with decreases GSIS. Similar to our findings, Paulmann et al. [12] showed a decrease in GSIS after 5HT pre-incubation in MIN6 cells as well as in rat β-cell lines INS1 and RINm5f. Interestingly, they report that 5HTP pre-incubation increases GSIS in RINm5f cells, suggesting differential actions for intracellular and extracellular 5HT pools on insulin secretion. Nonetheless, these authors do not show nor mention the effects of 5HTP incubation on GSIS in MIN6 cells.

In this study, we found that MIN6 cells and mouse pancreatic islets express Htr2b 5HT receptor. Interestingly, in islets of *db/db* mice, a model of diabetes and β-cells dysfunction [27], the mRNA level of Htr2b was ∼3-fold higher compared to islets of WT mice. We also found that prolonged incubation with extracellular 5HT or a selective Htr2b agonist reduces GSIS. On the contrary, pharmacological antagonism of Htr2b prevents 5HT inhibitory actions on GSIS, suggesting that prolonged activation of this receptor mediates serotoninergic inhibition of insulin secretion. Of note, the inhibitory effect of 5HT is less potent than the one exerted by Htr2b agonist, BW723C86. Importantly, although the physiological and pathological implications of 5HT on GSIS still remain debated, the inhibitory actions of extracellular 5HT on insulin secretion are consistent finding in the literature [5, 11–13, 35–37]. It is possible that, by itself, 5HT actions have a low functional impact on GSIS, but in combination with other dysregulations present in the diabetic milieu, could have a relevant pathological meaning. For example, we have found that Htr2b protein expression tend to be up-regulated by its own activation in MIN6 cells as well as others have shown that its mRNA levels are regulated by its own activation or inhibition in INS1(832/13) cells [7], suggesting that pharmacologic interventions on specific 5HT receptors may represent a clinically relevant therapeutic innovation in diabetes.

At the mechanistic level, we found that prolonged Htr2b activation in MIN6 cells decreases glucose-induced ATP content and basal and ATP-coupled mitochondrial respiration without modification in mitochondrial DNA copy number, mitochondrial superoxide content as well as no changes in mRNA levels of UCP2 and some genes of complex I, II and IV. All these facts led us to propose that prolonged Htr2b activation reduces GSIS by decreasing mitochondrial function and ATP production but without changing in mitochondrial uncoupling, biogenesis and superoxide production.

On the other hand, we found that prolonged Htr2b activation in MIN6 cells decrease and increase the mRNA levels of ATP synthase complex genes Atp4a and Atp5a1, respectively. These findings altogether with reduced basal and ATP-coupled OCR and glucose-stimulated ATP content, suggest that prolonged Htr2b activation leads to defects in ATP synthase activity. In line with this proposition, Htr2b overexpression in mice cardiomyocytes has been associated with reduced adenine nucleotide translocator and abnormal mitochondrial function [21].

Increased PGC1α expression appears to determine different metabolic outcomes depending on what specific tissue is targeted [38]. In adipose tissue, high PGC1α expression participates in induced-browning and activation of thermogenesis increasing the uncoupling mitochondrial respiration [38], while in skeletal muscle and myocardial tissues, PGC1α upregulation results in gene reprograming from glycolytic to glucose and fatty acids oxidative metabolism which is accompanied by enhancement of mitochondrial biogenesis and respiration as well as ATP production [38]. By contrast, in liver and pancreas PGC1α activation promotes hepatic glucose production and inhibits GSIS, respectively [38]. Here we found that prolonged Htr2b activation increases PGC1α and PPARy levels and that PGC1α overexpression blunts GSIS in MIN6 cells. Our results agree with the proposed role of PGC1α on suppression of β-cell energy metabolism and GSIS. In fact, overexpression of PGC1α to levels similar to those present in islets of diabetics rodents leads to a marked decreases of GSIS as consequences of alterations in expression of genes that participates in glucose sensing and metabolism and reduces glucose-induced ATP production in rat pancreatic islets [19]. These reported roles of PGC1α in β-cells are coherent with previous studies showing higher PGC1α levels in pancreatic islets of diabetic mice [19] as with the association between specific haplotypes in PPARGC1A, the gene encoding PGC1α, with indices of beta cell function in humans [39].

Elevated glucocorticoids (GCs) during the fetal period promote pancreatic β-cell dysfunction in the adulthood, possibly by a GCs-dependent elevation of PGC1α levels and secondary down-regulation of pancreatic duodenal homebox 1 (Pdx1) in β-cells [18]. Notably, the forced overexpression of PGC1α in β-cells during the adulthood fails to induce β-cell dysfunction [18]. These findings suggest that elevated levels of PGC1α in β-cells are possibly associated with alterations in β-cells development during fetal stages rather than with primary defects in GSIS in terminally differentiated β-cells. The implications *in vivo* of our observed deleterious effects of Htr2b and PGC1α-dependent reduction of GSIS in MIN6 cells, remain to be determined.

Previous studies have shown that extracellular 5HT reduces GSIS in different clonal β-cell lines as well as in mouse and rat pancreatic islets [5, 11–13, 35–37]. Importantly, it has been reported that 5HT perfusion *in vivo* is associated with decreased insulin plasma levels in mice [12, 35]. In humans, 5HT plasma levels are reduced after glucose intake [40], suggesting a functional link between circulating 5HT, GSIS and glucose homeostasis. This idea is further supported by the fact that dietary-induced obese mice have seven times higher serum 5HT than lean mice [41] and that plasma levels of 5HT and 5HT metabolite 5-hydroxyindole-3-acetic acid are elevated in diabetic subjects [42, 43]. Furthermore, there are reported cases of glucose intolerance and low insulin levels in patients with carcinoid syndrome, characterized by excessive systemic 5HT levels [44, 45].

Only one previous study has reported GSIS inhibition by a specific 5HT receptor [13]. After finding that islets from *db/db* have increased level of Htr2c, Zhang et al. showed that pharmacological activation of Htr2c was associated with decreased GSIS in MIN6 cells [13]. In our studies, Htr2c was undetectable in both MIN6 cells and mice pancreatic islets. We do not know the reason for this discrepancy, however, others have also failed to find Htr2c in human and mouse islets or in MIN6 cells [3, 7]. Here we report that Htr2b is present in MIN6 cells at the mRNA and protein level, that its protein level tend to be up-regulated by its own activation in MIN6 cells and that its mRNA is higher in islets of *db/db* in comparison with WT mice. In agreement with us, it was recently reported that Htr2b is expressed in mouse and human islets and, interestingly, its expression level is regulated by its own pharmacological activation or inhibition [7].

Our assertion that prolonged activation of Htr2b decreases GSIS is grounded on pharmacological evidence. It is unlikely that BW723C86 may activate other Htr2 subtypes in the concentrations we used in this study. In fact, BW723C86 affinity is three hundred times higher for Htr2b than for Htr2a. In addition, we found that Htr2a and Htr2c were not expressed in MIN6 cells and mouse pancreatic islets. In spite of that, the direct assessment of the actions of BW723C86 in islets of Htr2b deficient mice will provide conclusive functional evidence of the role of Htr2b on GSIS.

Bennet et al. recently showed that simultaneous Htr2b activation and glucose stimulation leads to increased insulin secretion by β-cells [7]. That study is, at the first glance, contradictory with our results; however, we believe that these discrepancies might result from the different duration of Htr2b activation between the two studies. In fact, equivalent dual biological effects have been reported for another Gα_q_-coupled receptor, the M3 cholinergic receptor [46]. In rat islets, short term co-stimulation with glucose and Carbachol increased GSIS in comparison with glucose-stimulation only. By contrast, prolonged M3 activation (3.5 hours) with Carbachol followed by glucose stimulation, virtually abolished GSIS [46]. Interestingly, it has been reported that pretreatment of rat islets with 5HT for 3 hours also impairs GSIS by altering glucose-induced activation of PLC activity [11].

**Fig 6:**
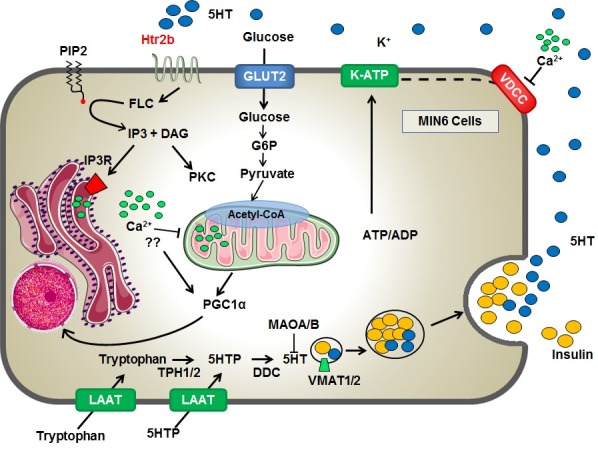
Proposed model for the inhibitory effect of prolonged Htr2b activation on GSIS in MIN6 cells. MIN6 cells constitute a functional microserotoninergic system, with the ability to synthesis 5HT from its precursors (tryptophan or 5HTP), storage, degrade and release this monoamine. The extracellular 5HT may decrease GSIS through the activation of the Htr2b receptor, the most abundantly 5HT receptor expressed in MIN6 cells. Prolonged Htr2b activation is accompanied with calcium release from endoplasmic reticulum and its inhibitory effects on GSIS is likely the result of enhanced PGC1α/PPARy expression and mitochondrial dysfunction with decreased glucose-stimulated ATP synthesis.

In conclusion, in this study we show that prolonged Htr2b activation with BW723C86 reduces GSIS in MIN6 cells possibly as a result of impaired mitochondrial activity and ATP production by mechanisms likely dependent on enhanced PGC1α/PPARy levels (Fig 6).

## Supporting Information

S1 Fig Htr2b expression in MIN6 cells after prolonged Htr2b activation. MIN6 cells were treated for 24 hours with vehicle or BW723C86 (10 μM) and then, Htr2b and Gapdh protein expression was evaluated by immunoblot. Graph bars represent means ± SEM of relative expression of Htr2b/Gapdh in treated versus control cells in three independent experiments. Student’s t test was used for statistical analysis.
(DOCX)

S2 Fig Calcium signals in MIN6 cells acutely stimulated with Htr2b agonist. MIN6 cells were cultured in standard conditions and charged with the calcium sensitive fluorescence dye Fluo4-AM (5 μM) in KRH buffer (0 mM glucose) by 30 min. The fluorescence signal (488 nm) was registered during 800 seconds by a confocal microscope (Carl Zeiss, Axiovert 200, LSM 5 Pascal) measured before and after the acute stimulation with (A) glucose (20 mM) and Carbachol (30 μM) or (B) the Htr2b selective agonist BW723C86 (10 μM). (C) The area under the curve (AUC) of fluorescence intensity adjusted by time was calculated before (white bars) and after (black bars) acute stimulus. The symbol ** denotes p<0.01 and *** p<0.001 in a Student’s t tests.
(DOCX)

S3 Fig Prolonged activation of Htr2b reduces GSIS in mouse pancreatic islets. Pooled islets from WT mice were treated with 50 μM 5HT or 10 μM BW723C86 for 6 h and then subjected to GSIS procedures. The GSIS assays were carry out with KRH buffer 2.8 (white bars) or 16.7 mM glucose (black bars). Concentration of insulin accumulated over 1 hour was measured by ELISA. Bars represent the mean of a single experiment (5 islets/well and 4 wells/condition), for that reason no statistical analysis was done. Number over the bars represents the stimulation index (SI).
(DOCX)

S4 Fig Prolonged Htr2b activation increases PGC1α levels in MIN6 cells. MIN6 cells were treated for 24 hours with BW723C86 (10 μM) and then the cytosolic and nuclear fractions were separated. PGC1α, Histone H3 (nuclear marker) and Gapdh (cytosolic marker) proteins were quantified by immunoblot.
(DOCX)

S5 Fig PGC1α isoforms levels in MIN6 cells and mouse islets. Total RNA was extracted from MIN6 cells (A) or pooled mouse pancreatic islets (n = 11 for WT and n = 12 for *db/db* mice) (B) with standard procedures. The mRNA levels of total PGC1α and its four isoforms were quantified by RT-qPCR and expressed as the fold change of 2^−ΔCt^ of isoforms vs. total PGC1α (2^−ΔCt isoform^/2^−ΔCt total^). The graph bars represent mean ± SEM of three independent experiments for MIN6 cells (A) and the unique values for mRNA expression in mice islets (B). Numbers over the bars correspond to mean Ct value.
(DOCX)

S6 Fig mRNA expression of mitochondrial complex genes in MIN6 cells after Htr2b prolonged activation. MIN6 cells were treated for 6 h with the Htr2b selective agonist BW723C86 (10 μM) or vehicle. Then, total RNA was extracted from MIN6 cells and the relative mRNA levels of mitochondrial complex genes were quantified by RT-qPCR and expressed as fold-change of 2^−ΔCt^ in treated vs. control cells (2^−ΔCt treated^/2^−ΔCt control^). Numbers over bars correspond to mean Ct values in control condition. The graph bars represent mean ± SEM of three independent experiments.
(DOCX)

S1 Table List of primers used to evaluate the mRNA expression of PGC1α isoforms, PPARy and mitochondrial complexes genes. (DOCX)

S2 Table 5HT receptors mRNA levels in pancreatic islets of WT and *db/db* mice. Total RNA was extracted from a pool of 335 islets from 11 WT mice or 312 islets from 12 *db/db* mice. 5HT receptors mRNA level was expressed as specific Ct values for every gene in WT and *db/db* mice islets and as fold-change of *db/db* to WT mice (2^−ΔCt *db/db*^/2^−ΔCt WT^). Data correspond to uniplicate values derived from pooled mouse islets.
(DOCX)
