# Supplementary figures and images for: Prolonged Activation of the Htr2b Serotonin Receptor Impairs Glucose Stimulated Insulin Secretion and Mitochondrial Function in MIN6 Cells
Source: PLoS One. 2017 Jan 27;12(1):e0170213. doi: 10.1371/journal.pone.0170213 (PMC5271329; doi:10.1371/journal.pone.0170213)

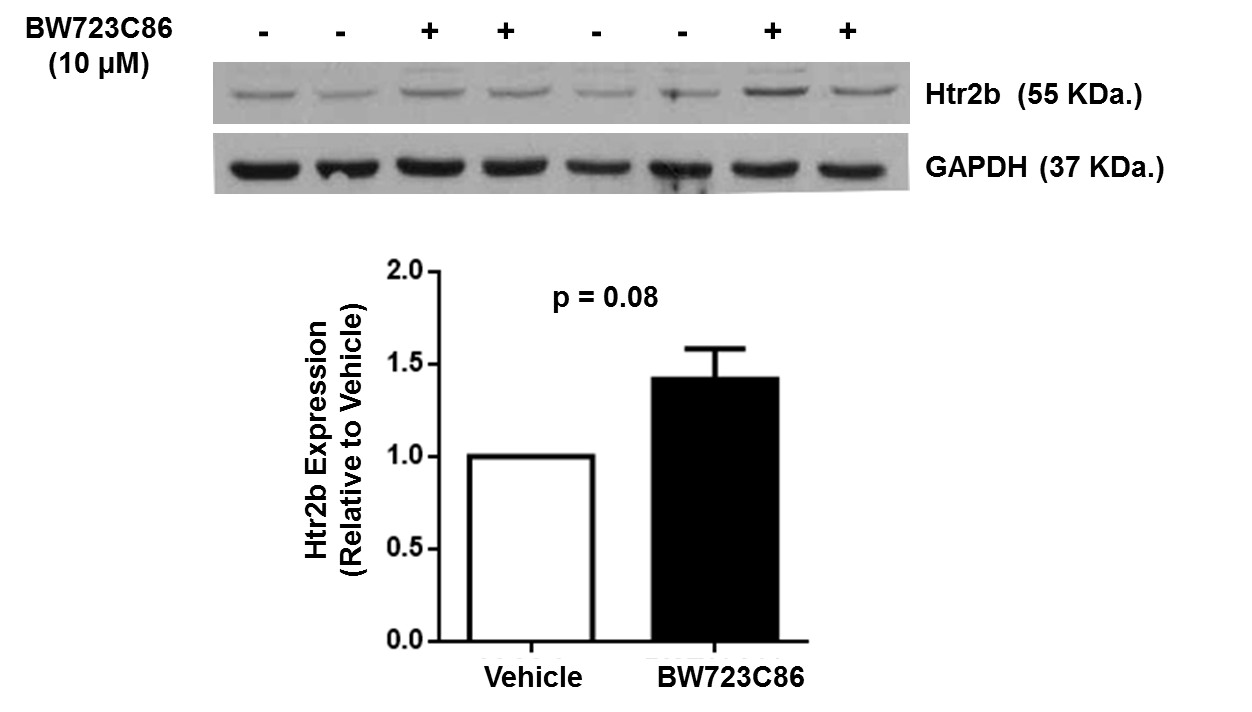

Supplement: S1 Fig — MIN6 cells were treated for 24 hours with vehicle or BW723C86 (10 μM) and then, Htr2b and Gapdh protein expression was evaluated by immunoblot. Graph bars represent means ± SEM of relative expression of Htr2b/Gapdh in treated versus control cells in three independent experiments. Student’s t test was used for statistical analysis. (DOCX) [file pone.0170213.s001.docx]
