# Supplementary material for: Prolonged Activation of the Htr2b Serotonin Receptor Impairs Glucose Stimulated Insulin Secretion and Mitochondrial Function in MIN6 Cells
Source: PLoS One. 2017 Jan 27;12(1):e0170213. doi: 10.1371/journal.pone.0170213 (PMC5271329; doi:10.1371/journal.pone.0170213)
Supplement: S1 Table — (DOCX) [file pone.0170213.s007.docx]

| **Primer** | **Sense** | **Sequence (5´-3´)** |
| --- | --- | --- |
| PGC1α total | Forward | TGATGTGAATGACTTGGATACAGACA |
|  | Reverse | GCTCATTGTTGTACTGGTTGGATATG |
| PGC1α1 | Forward | GGACATGTGCAGCCAAGACTCT |
|  | Reverse | CACTTCAATCCACCCAGAAAG CT |
| PGC1α2 | Forward | CCACCAGAATGAGTGACATGGA |
|  | Reverse | GTTCAGCAAGATCTGGGCAAA |
| PGC1α3 | Forward | AAGTGAGTAACCGGAGGCATTC |
|  | Reverse | TTCAGGAAGATCTGGGCAAAGA |
| PGC1α4 | Forward | TCACACCAAACCCACAGAAA |
|  | Reverse | CTG GAA GATATG GCA CAT |
| ndufa1 | Forward | ATGTGGTTCGAGATTCTCCCT |
|  | Reverse | TGGTACTGAACACGAGCAACT |
| sdha | Forward | GGAACACTCCAAAAACAGACCT |
|  | Reverse | CCACCACTGGGTATTGAGTAGAA |
| Cox5a | Forward | ATGCCTGGGAATTGCGTAAAG |
|  | Reverse | TGCGAACAGCACTAGCAAAAT |
| ATP4a | Forward | GCCAGCGTGGGTATCATCTC |
|  | Reverse | GCTGACAACTCTCCACAATCA |
| Atp5a1 | Forward | TCTCCATGCCTCTAACACTCG |
|  | Reverse | CCAGGTCAACAGACGTGTCAG |
| PPARγ | Forward | CCAGAGCATGGTGCCTTCGCT |
|  | Reverse | CAGCAACCATTGGGTCAGCTC |
