# Supplementary material for: Prolonged Activation of the Htr2b Serotonin Receptor Impairs Glucose Stimulated Insulin Secretion and Mitochondrial Function in MIN6 Cells
Source: PLoS One. 2017 Jan 27;12(1):e0170213. doi: 10.1371/journal.pone.0170213 (PMC5271329; doi:10.1371/journal.pone.0170213)
Supplement: S2 Table — Total RNA was extracted from a pool of 335 islets from 11 WT mice or 312 islets from 12 db/db mice. 5HT receptors mRNA level was expressed as specific Ct values for every gene in WT and db/db mice islets and as fold-change of db/db to WT mice (2−ΔCt db/db/2−ΔCt WT). Data correspond to uniplicate values derived from pooled mouse islets. (DOCX) [file pone.0170213.s008.docx]

| **Gene** | **Name** | **Islets CTs in** | | **Fold Change** |
| --- | --- | --- | --- | --- |
|  |  | **WT** | ***db/db*** |  |
| Htr1a | 5-hydroxytryptamine receptor 1A | 34.0 | >35 | -- |
| Htr1b | 5-hydroxytryptamine receptor 1B | 31.8 | 30.2 | 3.81 |
| Htr1d | 5-hydroxytryptamine receptor 1D | 33.4 | 33.0 | 1.62 |
| Htr1f | 5-hydroxytryptamine receptor 1F | 34.6 | >35 | -- |
| Htr2a | 5-hydroxytryptamine receptor 2A | 32.9 | >35 | -- |
| Htr2b | 5-hydroxytryptamine receptor 2B | 30.6 | 29.1 | 3.63 |
| Htr2c | 5-hydroxytryptamine receptor 2C | >35 | >35 | -- |
| Htr3a | 5-hydroxytryptamine receptor 3A | 28.7 | 34.6 | 0.02 |
| Htr3b | 5-hydroxytryptamine receptor 3B | 32.1 | 34.6 | 0.22 |
| Htr4 | 5-hydroxytryptamine receptor 4 | >35 | >35 | -- |
| Htr5a | 5-hydroxytryptamine receptor 5A | >35 | >35 | -- |
| Htr6 | 5-hydroxytryptamine receptor 6 | >35 | >35 | -- |
| Htr7 | 5-hydroxytryptamine receptor 7 | 34.7 | >35 | -- |
