# Supplementary material for: Prolonged Activation of the Htr2b Serotonin Receptor Impairs Glucose Stimulated Insulin Secretion and Mitochondrial Function in MIN6 Cells
Source: PLoS One. 2017 Jan 27;12(1):e0170213. doi: 10.1371/journal.pone.0170213 (PMC5271329; doi:10.1371/journal.pone.0170213)
Supplement: Supplementary file 4 [file pone.0170213.s002.docx]

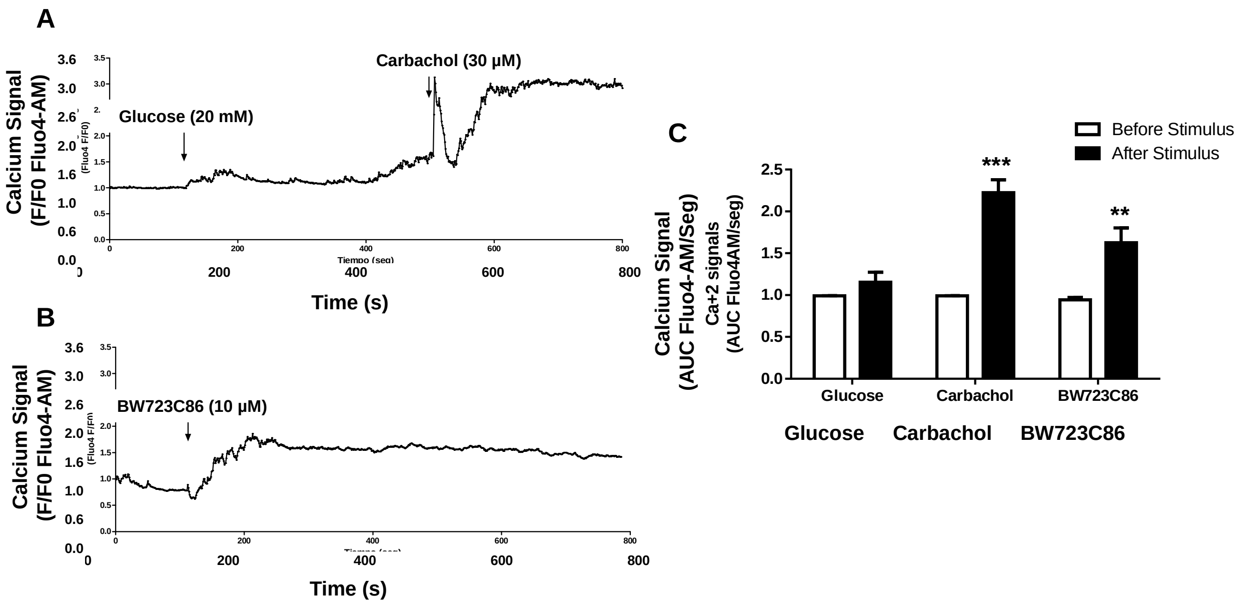
A
Calcium Signal (F/F0 Fluo4-AM)
3.6
3.0
2.6
2.0
1.6
1.0
0.6
0.0
Glucose (20 mM)
Carbachol (30 μM)
200
400
600
800
Time (s)
B
Calcium Signal (F/F0 Fluo4-AM)
BW723C86 (10 μM)
0
200
400
600
800
Time (s)
C
Before Stimulus
After Stimulus
Calcium Signal (AUC Fluo4-AM/Seg)
Ca+2 signals (AUC Fluo4AM/seg)
2.5
2.0
1.5
1.0
0.5
0.0
***
**
Glucose
Carbachol
BW723C86
Glucose Carbachol BW723C86
