# Supplementary material for: Prolonged Activation of the Htr2b Serotonin Receptor Impairs Glucose Stimulated Insulin Secretion and Mitochondrial Function in MIN6 Cells
Source: PLoS One. 2017 Jan 27;12(1):e0170213. doi: 10.1371/journal.pone.0170213 (PMC5271329; doi:10.1371/journal.pone.0170213)
Supplement: Supplementary file 5 [file pone.0170213.s003.docx]

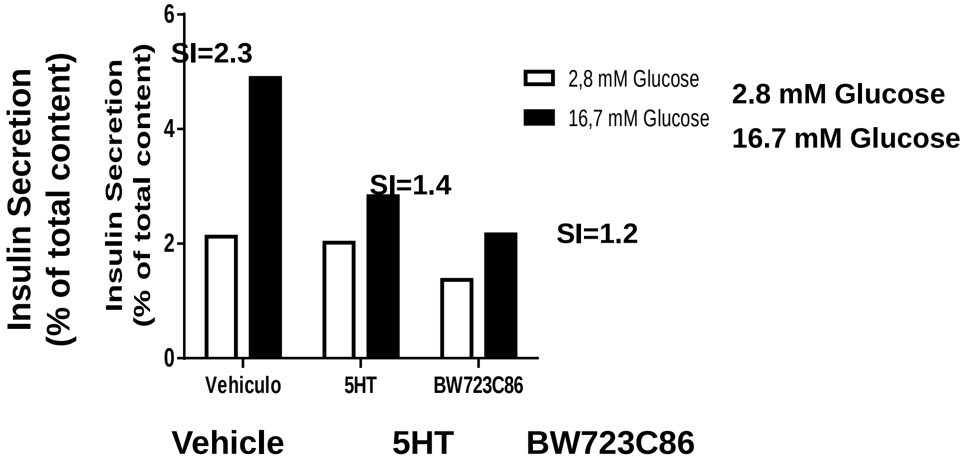
Insulin Secretion
(% of total content)
Insulin Secretion
(% of total content)
SI=2.3
SI=1.4
SI=1.2
2,8 mM Glucose
16,7 mM Glucose
2.8 mM Glucose
16.7 mM Glucose
0
2
4
6
Vehiculo
5HT
BW723C86
Vehicle
5HT
BW723C86
