# Supplementary material for: Prolonged Activation of the Htr2b Serotonin Receptor Impairs Glucose Stimulated Insulin Secretion and Mitochondrial Function in MIN6 Cells
Source: PLoS One. 2017 Jan 27;12(1):e0170213. doi: 10.1371/journal.pone.0170213 (PMC5271329; doi:10.1371/journal.pone.0170213)
Supplement: Supplementary file 6 [file pone.0170213.s004.docx]

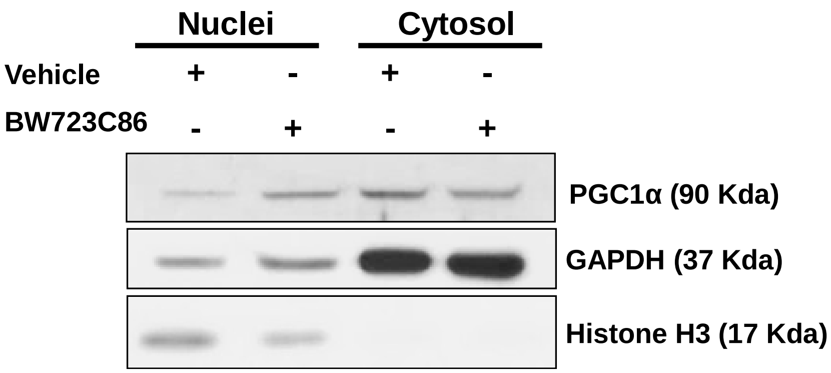
Nuclei
Cytosol
Vehicle + - + -
BW723C86 - + - +
PGC1α (90 Kda)
GAPDH (37 Kda)
Histone H3 (17 Kda)
