# Supplementary material for: Prolonged Activation of the Htr2b Serotonin Receptor Impairs Glucose Stimulated Insulin Secretion and Mitochondrial Function in MIN6 Cells
Source: PLoS One. 2017 Jan 27;12(1):e0170213. doi: 10.1371/journal.pone.0170213 (PMC5271329; doi:10.1371/journal.pone.0170213)
Supplement: Supplementary file 7 [file pone.0170213.s005.docx]

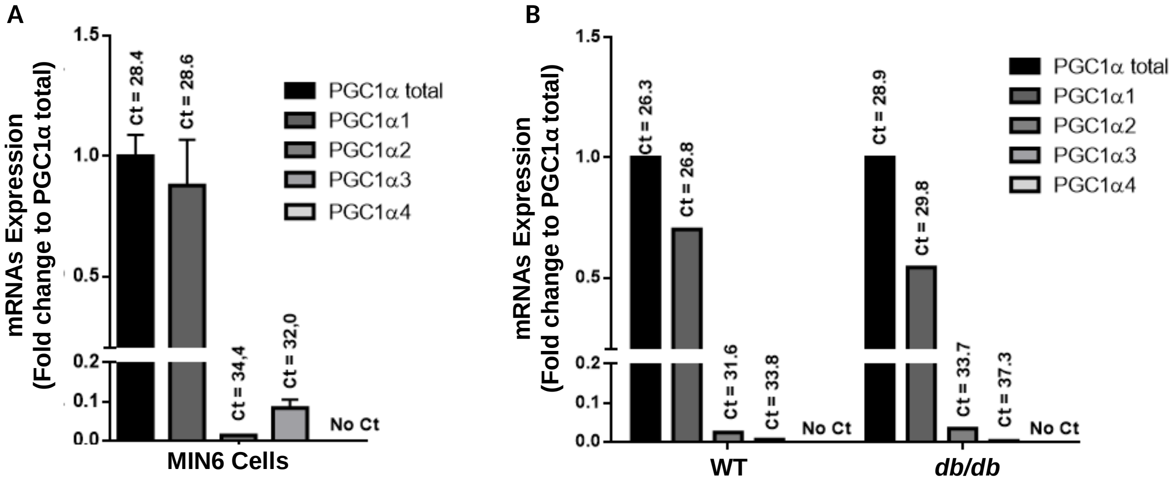
A
B
mRNAs Expression
(Fold change to PGC1α total)
1.5
1.0
0.5
0.2
0.1
0.0
Ct = 28.4
Ct = 28.6
Ct = 34,4
Ct = 32,0
No Ct
PGC1α total
PGC1α1
PGC1α2
PGC1α3
PGC1α4
MIN6 Cells
Ct = 26.3
Ct = 26.8
Ct = 31.6
Ct = 33.8
No Ct
Ct = 28.9
Ct = 29.8
Ct = 33.7
Ct = 37.3
No Ct
WT
db/db
