# Supplementary material for: Prolonged Activation of the Htr2b Serotonin Receptor Impairs Glucose Stimulated Insulin Secretion and Mitochondrial Function in MIN6 Cells
Source: PLoS One. 2017 Jan 27;12(1):e0170213. doi: 10.1371/journal.pone.0170213 (PMC5271329; doi:10.1371/journal.pone.0170213)
Supplement: Supplementary file 8 [file pone.0170213.s006.docx]

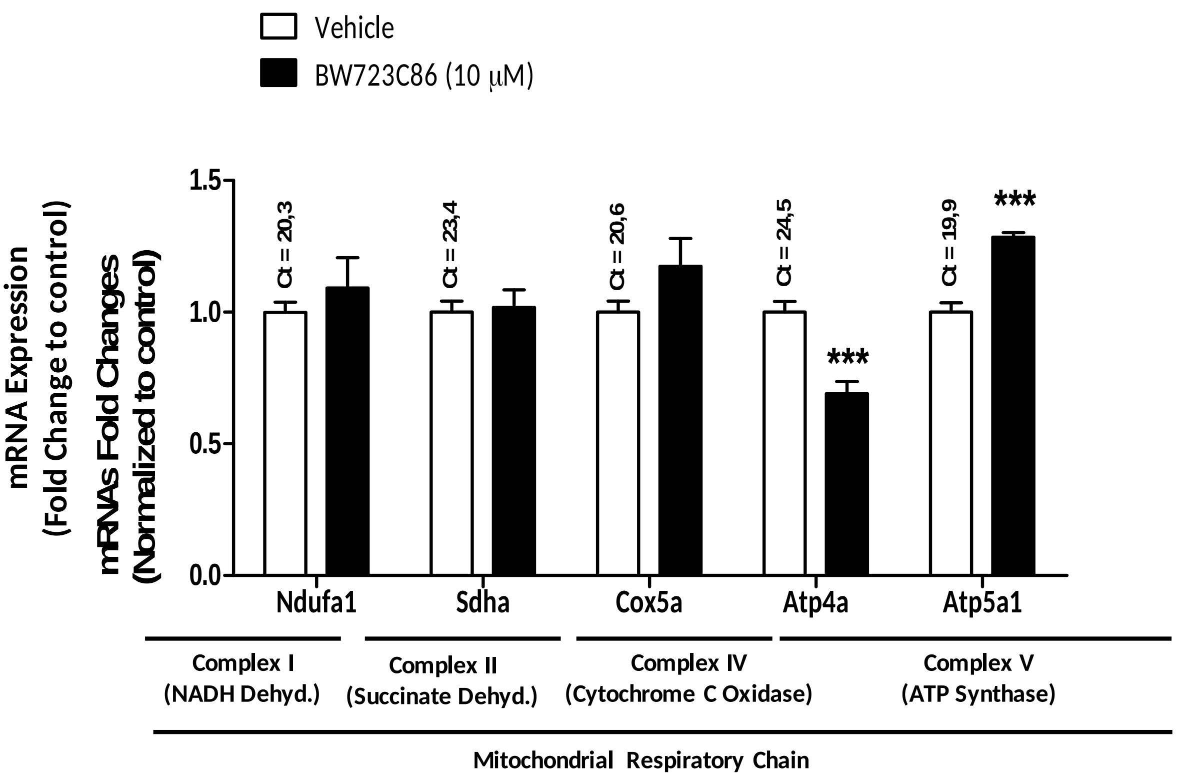
Vehicle
BW723C86 (10 μM)
mRNA Expression
(Fold Change to control)
mRNAs Fold Changes
(Normalized to control)
1.5
1.0
0.5
0.0
Ct = 20,3
Ct = 23,4
Ct = 20,6
Ct = 24,5
Ct = 19,9
***
***
Ndufa1
Sdha
Cox5a
Atp4a
Atp5a1
Complex I
(NADH Dehyd.)
Complex II
(Succinate Dehyd.)
Complex IV
(Cytochrome C Oxidase)
Complex V
(ATP Synthase)
Mitochondrial Respiratory Chain
